# An assessment of the suitability of online blood alcohol concentration calculators

**DOI:** 10.13075/ijomeh.1896.02802

**Published:** 2026

**Authors:** Joanna Stragierowicz, Natalia Jońska, Gabriela Szydłowska, Weronika Raczyńska, Anna Kilanowicz

**Affiliations:** Medical University of Lodz, Faculty of Pharmacy, Department of Toxicology, Łódź, Poland

**Keywords:** blood alcohol concentration, alcohol concentration, BAC calculator, BAC counter, online breath analyzer, virtual breath analyzer

## Abstract

**Objectives:**

The increasing popularity of online blood alcohol concentration (BAC) calculators which estimate alcohol levels based on user-entered variables such as sex, weight, and alcohol consumption data raises concerns about their reliability and their influence on the decision to drive. The aim of this study is to assess the accuracy and usability of 36 online BAC calculators in Polish and English.

**Material and Methods:**

The calculated BAC values were compared with reference values obtained from 8 female, 8 male in both fasted and post-meal states. Blood alcohol concentration was evaluated at the predicted maximum value and at 60 min and 120 min post-consumption.

**Results:**

Significant variability was noted: mean values exceeded reference concentrations by 39–49%, and few calculators correlated with reference values. Only the BACscan, Aisko, and Website No. 3 calculators yielded results comparable to reference values under certain conditions (Passing-Bablok regression and Bland-Altman plots). Furthermore, in the present study an online survey (N = 103) found that although 73.2% of users had used an online breathalyzer, 85.4% of these did not base decisions (e.g., driving) on their results, believing them to be unreliable.

**Conclusions:**

Overall, most online BAC calculators lack sufficient precision and are unsuitable for assessing intoxication. These findings underscore the need for public education on the limitations of such tools and call for better regulation of their accuracy and presentation.

## Highlights

- Most online blood alcohol concentration (BAC) calculators significantly overestimate blood alcohol levels.
- User-entered data is often insufficient for accurate BAC prediction.
- Public survey shows high usage but low trust in online breath analyzers.
- Online BAC tools are unreliable for safety-critical decisions, like driving.

## INTRODUCTION

According to the European Transport Safety Council, there were >20 000 deaths on European Union (EU) roads each year in 2022 and 2023 alone [1]. The risk of an accident, and its severity, dramatically increase with the blood ethanol content of the driver. Drunk driving is a leading cause of fatal accidents: around 25% of all road deaths in Europe are estimated to be related to intoxication [1]. As such, drivers involved in road traffic accidents are routinely checked for blood alcohol concentration (BAC), as are any suspected of being intoxicated.

Ethyl alcohol has a direct impact on the central nervous system (CNS), deteriorating the cognitive, visual, and motor functions and impairing driving skill [2]. The ability to keep a vehicle in motion, reaction time, perception, and attention span begin to weaken with a BAC as low as 0.2 g/l [3]. It has been estimated that a driver with a BAC 0.2–0.4 g/l is at a 1.4-times greater risk of a fatal accident than a sober driver; this risk is further increased 11.1 times by a BAC 0.5–0.9 g/l [4]. The legal limits defining the permissible BAC for private and professional drivers in European countries are given in Table 1.

**Table 1:** Blood alcohol concentration (BAC) limits in Europe [5]

Country	BAC [g/l]		
	standard	drivers	
		commercial	novice
Czech Republic, Hungary, Romania, Slovakia	0.0	0.0	0.0
Estonia, Poland, Sweden, Norway	0.2	0.2	0.2
Lithuania	0.4	0.0	0.0
Croatia, Germany, Italy, Slovenia	0.5	0.0	0.0
Austria, Switzerland	0.5	0.1	0.1
Belgium	0.5	0.2	0.5
Cyprus, Greece, Ireland, Luxembourg, Malta, Portugal	0.5	0.2	0.2
Spain	0.5	0.3	0.3
Latvia, France, Netherlands	0.5	0.5	0.2
Bulgaria, Denmark, Finland, Scotland	0.5	0.5	0.5
United Kingdom	0.8	0.8	0.8

According to the European Road Safety Observatory Safety Performance Indicator (ERSO SPI) report [6], countries with BAC limits of ≤0.2 g/l (including the Czech Republic, Hungary, Norway, Poland, and Sweden) are among those with the lowest drink-driving scores, which results in lower mortality rates compared to countries where the BAC limit is >0.2 g/l.

Interestingly, European countries with a drinking culture associated with wine production and consumption, such as Portugal and France, demonstrate greater alcohol consumption than countries with a beer-drinking tradition i.e., in Northern European [2]; this may be reflected in the number of fatal incidents caused by alcohol consumption.

**Figure 1.**
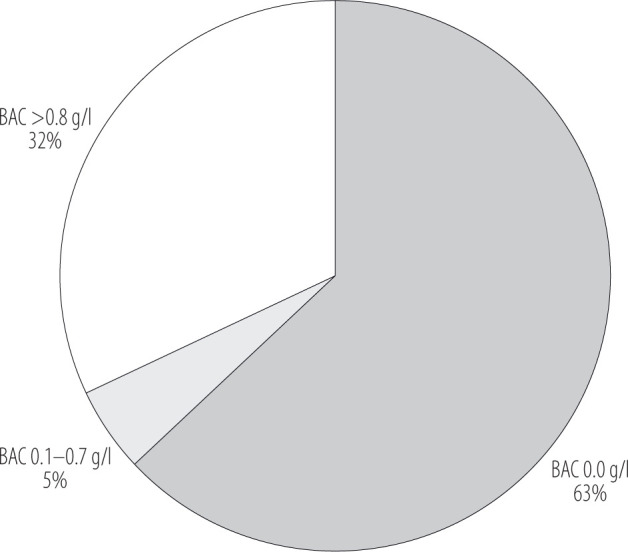
Distribution of blood alcohol concentrations (BACs) for drivers involved in fatal traffic crashes [8]

In contrast, the BAC limit in almost all states in the USA is 0.8 g/l. In 2022 in the USA, drink-driving drivers (i.e., with BAC >0.8 g/l) were responsible for a total of 13 524 fatalities, accounting for 32% of all road traffic fatalities [7] (Figure 1). In contrast, only 5% of those surveyed at the time of a traffic accident had a BAC >0.1 g/l but lower than the legal limit (0.8 g/l).

The concentration of ethanol in a driver is typically measured with a breath analyzer (done first), or by blood analysis. In Poland, both methods have the same evidentiary value in court cases. However, following numerous advertising campaigns, and with growing public awareness of the negative impact of drink-driving, various methods of self-assessment of sobriety are gaining popularity. These methods include breathalyzers for personal use. In such cases, International Organization of Legal Metrology recommendations specify that their measurement technologies should be described in the technical documentation provided by the manufacturer [9]. These electronic devices are widely available for purchase, are characterized by easy and intuitive operation and often reliable measurements, provided that they are regularly serviced and calibrated at authorized centers. Unfortunately, they are used only as a device for indicative monitoring of alcohol concentration, they are not all subject to expensive homologation (unlike police breathalyzers), and the results they produce have no evidentiary value in court cases.

However, the popularity of virtual breathalyzers is also growing. These are online calculators not covered by calibration or inspection which can be easily found on the internet by users who may not have experience in determining sobriety status. Such online calculators, known as BAC calculators, rely solely on data provided online by the user; this can include basic data such as the amount of alcohol consumed, the type of alcoholic beverage, the time since the start of alcohol consumption, user sex and weight, as well as height, age, body type, meals consumed and the subjectively assessed metabolic rate of alcohol.

While the data may be provided accurately by the user, and the calculator itself may be well furnished with beverage types of different strengths, using such a simple online application to determine sobriety status is regarded as unreliable and untrustworthy. As no in-depth studies supported by statistical analyses have been performed to date to determine the reliability on-line breathalyzers, the purpose of this work was to compare the accuracy of online BACs presented in Polish and English calculators based on a their statistical analysis.

## MATERIAL AND METHODS

### Study subjects

The subject of this study was the most popular online breathalyzers (in Polish and English) were selected based on Google searches under the keywords: “online breathalyzer,” “virtual breathalyzer,” “online alcomat,” “bac calculator,” “bac counter,” “wirtualny alkomat,” “alkomat online,” “kalkulator promili,” “alkomat internetowy,” “kalkulator alkoholowy.” From the initial searches, 42 calculators were extracted. Some of these were inactive and did not allow calculation. Finally, 36 calculators were included in the study (Table 2); all estimate the theoretical concentration of alcohol in the blood based on specific variables. Permission was given to include the names of the calculators was given by the website administrators. In the absence of permission to publish the name of the website in question, the data has been anonymized.

**Table 2:** List of blood alcohol concentration (BAC) calculators and their acronym names used in the study on the accuracy and usability of 36 online BAC calculators in Polish and English

Website	Acronim
https://aisko.pl/wirtualny-alkomat-aisko	Aisko
https://alcohol.org/bac-calculator/ ^a^	Alcohol.org
https://alkomat-online.pl/	Alkomat-online
https://alko-maty.pl/content/7-wirtualny-alkotest	Alko-maty
https://alkopatrol.pl/wirtualny-alkomat-online	Alkopatrol
https://alkotester.pl/wirtualny-alkomat/	Alkotester
https://bacscan.pl/alkomat-wirtualny/	BACscan
https://docelu.pl/wirtualny-alkomat	Docelu
https://odleglosci.info/wirtualny-alkomat	Odleglosci
https://paihdelinkki.fi/en/tests-and-counters/counters/bac-counter/	Paihdelinkki
https://www.abcalkoholu.pl/sprawdz-promile/sprawdz-online/	Abcalkoholu
https://www.alkomaty.biz/wirtualny-alkomat/index.php	Alkomaty.biz
https://www.blokadaalkoholowa.com/wirtualny-alkomat/	Blokadaalkoholowa
https://www.calculator.net/bac-calculator.html	Calculator.net
https://www.drinkdriving.org/bac-calculator.php	Drinkdriving
https://www.educalcool.qc.ca/en/tools/calcoholator/	Educalcool
https://www.ilemogewypic.pl/alkomat.php	Ilemogewypic
https://www.kazdypromil.pl/alkomat/	Kazdypromil
https://www.medme.pl/kalkulatory/alkohol	Medme
https://www.omnicalculator.com/pl/zdrowie/promile	Omnicalculator
https://www.originalabsinthe.com/alcohol-calculator#step-one	Originalabsinthe
https://www.rupissed.com/	Rupissed
https://wylecz.to/kalkulatory/kalkulator-alkoholu/	Wylecz.to
Website No. 1	Website No. 1
Website No. 2	Website No. 2
Website No. 3	Website No. 3
Website No. 4	Website No. 4
Website No. 5	Website No. 5
Website No. 6	Website No. 6
Website No. 7	Website No. 7
Website No. 8	Website No. 8
Website No. 9	Website No. 9
Website No. 10	Website No. 10
Website No. 11	Website No. 11
Website No. 12	Website No. 12
Website No. 13	Website No. 13

a The BAC calculator is meant for educational purposes only. The BAC calculator and information generated from it is not intended to replace the medical advice of your doctor or health care provider and should not be relied upon; nor do the BAC calculator or information generated from it constitute legal advice.

Different sets of data are needed for the websites, although most require sex, age, weight, height, body type, metabolic rate, meal consumption (fasting and post-meal consumption), amount and type of alcohol consumed, as well as the time of consumption. To evaluate the usability of the websites we tested, we used data from 16 individuals (8 women and 8 men) from the study Sekuła et al. [10] and treated these as reference values. The data used were: gender, height, body weight, the amount and concentration of alcohol consumed, and the results of BAC tests, defined as maximum concentration (C_max_) and obtained after 60 min and 120 min, both on an empty stomach and after a meal. Only 5 online breathalyzers (out of 36 analyzed) also required the user to enter their age. In such cases, a calculation model for ages 20 years, 40 years, and 60 years was used.

For calculators that did not have the option to enter the exact amount of pure alcohol consumed or the declared alcoholic beverage, the most approximate amount was calculated and then entered. For body type, the calculated BMI was used; for metabolism, “normal” was selected. The terms used to describe being fasted included: empty stomach/fasting/very hungry. In the case of alcohol consumption after a meal, the following were selected accordingly: full stomach/big meal/full.

The BAC calculators were used to determine the BAC values in g/l: C_max_ and also 60 and 120 min after the start of consumption. For websites that did not directly provide C_max_ values, the highest reported concentration was used in the analysis, regardless of the time (T_max_). Some calculators also present the dependence of BAC on time in graph form.

### Questionnaire surveys

In addition, an online survey was conducted among different age groups to assess the popularity and use of online breathalyzers. The survey participants were recruited using a convenience sampling approach. The questionnaire link was distributed online among university students and their family members via social networks and direct communication. Participation was voluntary and anonymous. The survey used the following questions:

– Sex (possible answers: man/woman);
– Age (answers in years);
– Are you familiar with the term “virtual breathalyzer”? (possible answers: yes/no). If “no,” end of the survey;
– If “yes” in question 3: Have you ever used an online breathalyzer? (possible answers: yes/no). If “no,” end of the survey;
– If “yes” in question 4: For what purpose did you enter your data into the online breathalyzer? (possible answers: out of curiosity/to verify my state of sobriety before driving a car/prospective calculus check – how much can I drink and what alcohol concentration would I have?/to compare myself with other people/others: ……………);
– If “yes” in question 4: How often do you use the online breathalyzer? (possible answers: occasionally/frequently/after any alcohol consumption/others: ……………);
– If “yes” in question 4: Do you make your decision to implement an action (e.g., driving) based on the results given by a breathalyzer? (possible answers: yes/no);
– If “yes” in question 4: What online breathalyzers do you use most frequently? (possible answers: appearing highest in the search list/appearing lower in the search list/I have my favourite/other: ……………);
– If “yes” in question 4: What features of a breathalyzer influence your decision to use it? (possible answers: number of data obtained/data to be entered/popularity/graphic layout/others: ……………);
– If “yes” in question 4: Which breathalyzers have you used? (possible answers: list of BAC calculators);
– If an accurate breathalyzer was selected in question 10: How would you rate the usefulness of the virtual breathalyzer you used? (possible answers: helpful/not useful/practical/useful/others: ……………);
– If an accurate breathalyzer was selected in question 10: How would you rate the reliability of the online breathalyzer you used? (Possible answers: authoritative/trustworthy/uncertain/suspicious/others: ……………).

### Statistical analysis

The statistical analysis was performed using Statistica software v. 13. The basic descriptive statistics included the calculation of the mean (M), median (Me), minimum value, maximum value, standard deviation (SD), and coefficient of variation (CV) for each breathalyzer, taking into account age and the time since alcohol consumption. Those on-line breathalyzers with a CV >2% were included in the analysis. When discussing the results obtained from breathalyzers, identical values were sometimes recorded for different websites. In such cases, they were treated as a single record.

The Shapiro-Wilk test was used to assess the normality of the data distribution. The correlation between variables was evaluated using the parametric Pearson's test, for normally-distributed variables, or the non-parametric Spearman's test, for non-normally-distributed variables. Additionally, the breathalyzers were subjected to a comparison test and usefulness method evaluation based on the Passing-Bablok regression. The non-parametric Mann-Whitney U test or the parametric Student's t-test for independent samples (with respect to groups) was used to analyze differences in the results of alcohol consumption according to the meal consumed. Due to the small sample size (N = 16), a test with independent variance estimation was used. The data was represented in graph form where appropriate.

## RESULTS

### General characteristic of online BAC calculators

The data required by each calculator are presented in Table 3. All calculators asked about sex and almost all (97.2%) included body weight. In contrast, much fewer included questions on personal characteristics, such as age (30.6%) or height (38.9%). Body type and metabolic rate were included even less frequently, with each being required by only 8.3% of cases.

**Table 3:** Data requested by the online blood alcohol concentration (BAC) calculators

BAC calculator	Data requested									
	sex	age	weight	height	body type	metabolism	time	volume of alcoholic beverage	concentration of alcoholic beverage	meal
Abcalkoholu	included	included	included	not included	not included	not included	only start	included	limited types	not included
Aisko	included	not included	included	not included	not included	not included	start to end	approximate amount	limited types	3-degree scale
Alcohol.org	included	not included	included	not included	not included	not included	only start	approximate amount	limited types	not included
Alko-maty	included	not included	included	not included	not included	not included	only end	approximate amount	limited types	4-degree scale
Alkomaty.biz/Website No. 7/Docelu	included	included	included	included	not included	not included	start to end	included	accurate concentration	4-degree scale
Alkotester/Website No. 8/Drinkdriving/Blokadaalkoholowa/Website No. 12	included	not included	included	not included	not included	not included	only end	approximate amount	limited types	not included
Aisko	included	not included	included	included	not included	not included	start to end	included	accurate concentration	not included
BACscan	included	not included	included	not included	not included	not included	start to end	approximate amount	limited types	4-degree scale
Calculator.net	included	not included	included	not included	not included	not included	only start	approximate amount	accurate concentration	not included
Ilemogewypic	included	not included	included	included	not included	not included	start to end	included	accurate concentration	3-degree scale
Kazdypromil/Educalcool/Omnicalculator	included	not included	included	not included	not included	not included	only start	included	accurate concentration	not included
Medme/Alkomat-online/Alkopatrol	included	included	included	included	included	included	start to end	included	accurate concentration	4-degree scale
Odleglosci	included	included	included	included	not included	not included	start to end	included	accurate concentration	3-degree scale
Originalabsinthe	included	not included	included	included	not included	not included	only start	included	accurate concentration	other scale
Rupissed	included	included	included	included	not included	not included	only start	included	accurate concentration	not included
Summary	included (100%)	included (30.6%)	included (97.2%)	included (38.9%)	included: underweight/normal/overweight (8.3%)	included: slow/normal/fast (8.3%)	time to start and end of drinking (44.4%); only time since start drinking (27.8%)	included: exact amount (66.7%)	limited types choices (38.9%); accurate concentration (58.3%)	4-degree scale: fasting/small meal/normal meal/big meal (8.3%); included 3-degree scale: empty stomach/half full stomach/full stomach (25.0%)
Website No. 1/Website No. 9/Paihdelinkki	included	not included	included	not included	not included	not included	start to end	included	accurate concentration	not included
Website No. 2	included	not included	included	not included	not included	not included	only end	included	accurate concentration	not included
Website No. 3	included	not included	included	included	not included	not included	start to end	included	accurate concentration	4-degree scale
Website No. 4	included	included	included	included	not included	not included	only end	included	limited types	other scale
Website No. 5	included	included	included	included	not included	not included	only end	approximate amount	limited types	not included
Website No. 10	included	not included	included	not included	not included	not included	start to end	approximate amount	limited types	not included
Website No. 11	included	not included	included	not included	not included	not included	only start	included	limited types	not included
Website No. 13	included	not included	included	not included	not included	not included	only start	included	not included	not included
Wylecz.to	included	not included	not included	not included	not included	not included	other scale	included	accurate concentration	not included

Not every given calculator also required data related to the alcohol consumed: the time when this took place, the volume and concentration consumed, and whether consumption was accompanied by a meal or an empty stomach. Regarding the time of alcohol consumption, almost half of the websites (44.4%) required both the time when drinking began and when it ended; in contrast, almost all of the remaining calculators only asked about the time when drinking began (27.8%) or only when it ended (25.0%). Only 1 online calculator used a different scale.

Regarding the amount (volume) of alcohol consumed, most websites required an exact value (66.7%), with the other calculators relying only on approximate values, such as the possibility of selecting only specific volumes of beers, wines or other alcoholic beverages. Similarly, most sites (58.3%) requested a specific alcohol concentration for the consumed beverage, while the remaining 38.9% based their calculations on approximate values, i.e., usually a list of specific concentrations; alternatively, 2.8% did not consider alcohol concentration at all.

Regarding fasting or fed state, most sites (61.1%) did not take into account the meal at all. However, 25.0% used a 3-step scale (empty stomach/half full stomach/full stomach), 8.3% used a 4-step scale (fasting/small meal/normal meal/big meal), and only 2 (5.6%) used a different scale.

### Calculations of predicted maximum BAC and at 60 min and 120 min after consumption using online breathalyzers

**Figure 2.**
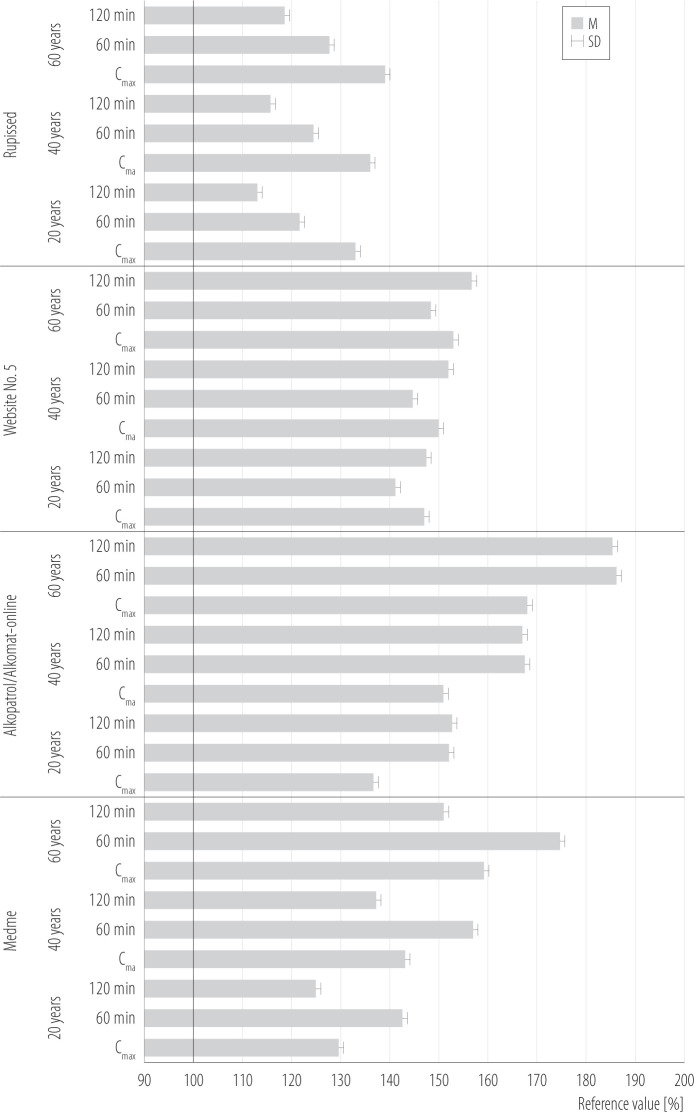
Comparison of maximum alcohol concentrations (C_max_), and alcohol concentrations at 60 min and 120 min after consumption between online calculators; the included sites incorporated age in the calculation and gave different results for various age groups (ages 20 years, 40 years and 60 years were used) [10]

**Figure 3.**
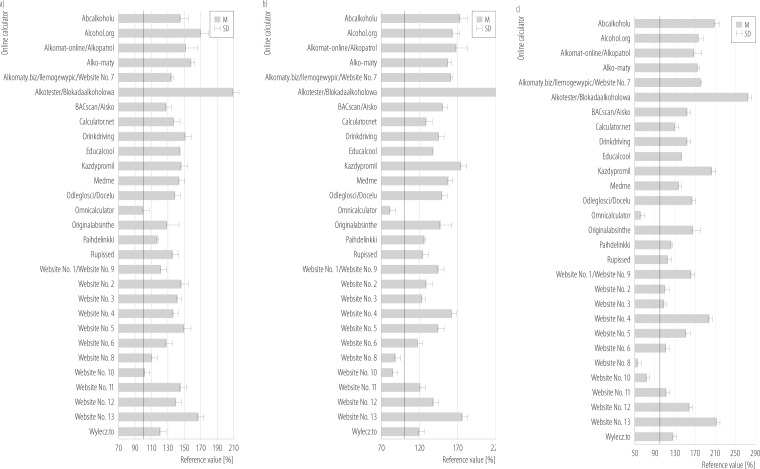
Comparison of on-line calculators with regard to the calculated blood alcohol concentrations (BAC): a) maximum (C_max_), b) 60 min after consumption, and c) 120 min after consumption [10]

**Figure 4.**
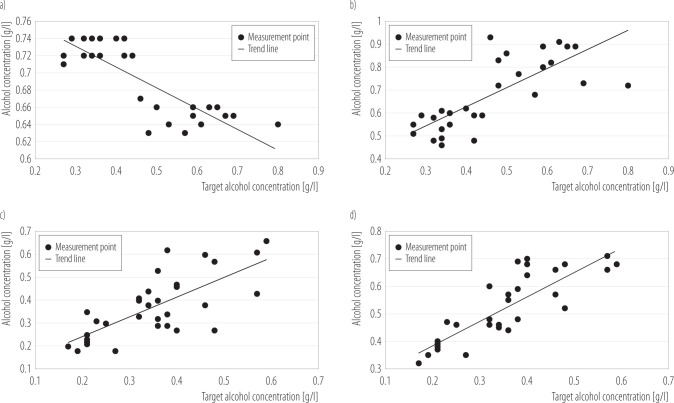
Relationship between the calculated results from the online blood alcohol concentrations (BAC) calculators and the reference values (i.e. target alcohol concentration) [10] after 60 min for a) Alkomatybiz, Ilemogewypic i Website No. 7 and b) BACscan and Aisko and for 120 min for c) Website No. 3 and d) BACscan and Aisko

The predicted maximum BAC and also after 60 min and 120 min after consumption calculated using the online breathalyzers are shown in Figures 2–4 and Tables 4. The results from the comparison with study Sekuła et al. [10], presented as percent of reference value, are given in Figure 2. Only 5 of the 36 evaluated websites took from online calculators age into account and gave different results depending on the age group. All of the results shown in Figure 2 are higher than the reference values given by Sekuła et al. [10], i.e., 113–186% of this value. In addition, the final result became more inflated as the entered age was increased (i.e., 20 years old, 40 years old, or 60 years old).

**Table 4:** Descriptive statistics derived from the calculated C_max_ blood alcohol concentration (BAC) results from each online breathalyzer, 60 min and 120 min after alcohol consumption and the reference value [10]

BAC calculator	Blood alcohol concentration																	
	C_max_						60 min after alcohol consumption						120 min after alcohol consumption					
	M [g/l]	Me [g/l]	min. [g/l]	max [g/l]	SD	CV [%]	M [g/l]	Me [g/l]	min. [g/l]	max [g/l]	SD	CV [%]	M [g/l]	Me [g/l]	min. [g/l]	max [g/l]	SD	CV [%]
Reference value	0.551	0.540	0.270	0.880	0.139	25.3	0.474	0.450	0.270	0.800	0.143	30.2	0.350	0.360	0.170	0.590	0.115	33.0
Abcalkoholu	0.751	0.755	0.730	0.780	0.016	2.1	0.751	0.755	0.730	0.780	0.016	2.1	0.650	0.655	0.620	0.690	0.023	3.5
Alcohol.org	0.881	0.900	0.700	1.000	0.103	11.7	0.713	0.700	0.600	0.800	0.079	11.1	0.556	0.550	0.400	0.700	0.108	19.3
Alkomat-online/Alkopatrol																		
20 years old	0.705	0.700	0.650	0.830	0.048	6.8	0.658	0.660	0.520	0.750	0.054	8.2	0.488	0.495	0.370	0.600	0.074	15.1
40 years old	0.778	0.755	0.690	0.970	0.076	9.7	0.724	0.730	0.550	0.880	0.075	10.4	0.531	0.535	0.420	0.640	0.067	12.6
60 years old	0.864	0.815	0.730	1.170	0.118	13.7	0.804	0.800	0.580	1.050	0.109	13.6	0.587	0.590	0.490	0.690	0.058	9.9
Alko-maty	0.815	0.815	0.700	0.910	0.063	7.8	0.685	0.690	0.550	0.800	0.067	9.7	0.555	0.560	0.400	0.690	0.075	13.6
Alkomaty.biz/Ilemogewypic/Website No. 7	0.689	0.690	0.630	0.740	0.043	6.2	0.689	0.690	0.630	0.740	0.043	6.2	0.557	0.560	0.490	0.620	0.044	7.8
Alkotester/Blokadaalkoholowa	1.069	1.050	0.690	1.450	0.245	22.9	0.949	0.930	0.570	1.330	0.245	25.8	0.829	0.810	0.450	1.210	0.245	29.6
BACscan/Aisko	0.689	0.650	0.460	0.930	0.155	22.5	0.689	0.650	0.460	0.930	0.155	22.5	0.518	0.480	0.320	0.710	0.123	23.8
Calculator.net	0.710	0.710	0.680	0.740	0.030	4.3	0.560	0.560	0.530	0.590	0.030	5.4	0.410	0.410	0.380	0.440	0.030	7.4
Drinkdriving	0.782	0.780	0.750	0.820	0.017	2.1	0.630	0.630	0.600	0.670	0.017	2.7	0.481	0.480	0.450	0.520	0.016	3.4
Educalcool	0.750	0.750	0.740	0.760	0.010	1.3	0.599	0.600	0.580	0.610	0.011	1.8	0.449	0.450	0.430	0.460	0.011	2.5
Kazdypromil	0.758	0.755	0.740	0.770	0.011	1.5	0.758	0.755	0.740	0.770	0.011	1.5	0.638	0.635	0.620	0.650	0.011	1.7
Medme																		
20 years old	0.669	0.665	0.620	0.780	0.044	6.6	0.618	0.620	0.500	0.690	0.045	7.2	0.404	0.410	0.250	0.550	0.098	24.2
40 years old	0.738	0.715	0.660	0.920	0.072	9.7	0.679	0.685	0.530	0.800	0.062	9.2	0.443	0.450	0.290	0.580	0.095	21.5
60 years old	0.819	0.775	0.700	1.100	0.109	13.3	0.755	0.760	0.560	0.960	0.092	12.1	0.485	0.495	0.330	0.620	0.090	18.6
Odleglosci/Docelu	0.716	0.710	0.700	0.730	0.009	1.3	0.648	0.650	0.630	0.660	0.008	1.2	0.511	0.505	0.490	0.530	0.014	2.7
Omnicalculator	0.514	0.510	0.510	0.520	0.005	1.0	0.354	0.355	0.340	0.370	0.013	3.6	0.194	0.195	0.170	0.220	0.022	11.4
Originalabsinthe	0.676	0.670	0.560	0.800	0.065	9.5	0.647	0.640	0.560	0.750	0.046	7.0	0.519	0.520	0.450	0.600	0.041	7.8
Paihdelinkki	0.606	0.600	0.500	0.700	0.044	7.2	0.550	0.550	0.500	0.600	0.051	9.2	0.388	0.400	0.300	0.500	0.061	15.7
Rupissed																		
20 years old	0.691	0.690	0.600	0.780	0.058	8.4	0.531	0.525	0.410	0.660	0.084	15.8	0.369	0.375	0.210	0.550	0.111	30.0
40 years old	0.708	0.715	0.600	0.810	0.071	10.0	0.543	0.555	0.410	0.690	0.094	17.3	0.379	0.390	0.210	0.570	0.119	31.4
60 years old	0.724	0.735	0.600	0.840	0.085	11.7	0.558	0.575	0.410	0.720	0.107	19.2	0.389	0.410	0.210	0.590	0.128	32.9
Website No. 1/Website No. 9	0.629	0.625	0.610	0.640	0.011	1.7	0.629	0.625	0.610	0.640	0.011	1.7	0.509	0.505	0.490	0.520	0.011	2.1
Website No. 2	0.758	0.760	0.740	0.770	0.010	1.3	0.562	0.555	0.520	0.630	0.035	6.3	0.351	0.335	0.200	0.480	0.076	21.7
Website No. 3	0.765	0.740	0.510	1.070	0.204	26.6	0.568	0.545	0.340	0.870	0.167	29.4	0.372	0.345	0.180	0.660	0.137	36.9
Website No. 4	0.709	0.710	0.660	0.780	0.033	4.6	0.705	0.700	0.650	0.780	0.035	4.9	0.595	0.615	0.410	0.780	0.124	20.8
Website No. 5																		
20 years old	0.760	0.750	0.630	0.940	0.077	10.1	0.610	0.600	0.480	0.790	0.077	12.7	0.460	0.450	0.330	0.640	0.077	16.8
40 years old	0.776	0.765	0.660	0.940	0.073	9.4	0.626	0.615	0.510	0.790	0.073	11.7	0.476	0.465	0.360	0.640	0.073	15.4
60 years old	0.792	0.775	0.690	0.950	0.071	8.9	0.642	0.625	0.540	0.800	0.071	11.0	0.492	0.475	0.390	0.650	0.071	14.3
Website No. 6	0.666	0.660	0.640	0.750	0.031	4.7	0.513	0.500	0.460	0.600	0.043	8.4	0.358	0.340	0.280	0.460	0.059	16.6
Website No. 8	0.578	0.555	0.440	0.790	0.111	19.2	0.391	0.395	0.230	0.730	0.133	34.1	0.188	0.230	0.030	0.420	0.135	71.8
Website No. 10	0.526	0.530	0.440	0.600	0.048	9.1	0.369	0.360	0.290	0.450	0.050	13.5	0.228	0.230	0.070	0.300	0.059	25.8
Website No. 11	0.753	0.755	0.730	0.780	0.019	2.5	0.528	0.505	0.380	0.640	0.084	15.8	0.356	0.360	0.150	0.490	0.117	33.0
Website No. 12	0.714	0.700	0.460	0.970	0.164	23.0	0.594	0.580	0.340	0.850	0.164	27.7	0.474	0.460	0.220	0.730	0.164	34.7
Website No. 13	0.864	0.860	0.850	0.880	0.015	1.7	0.764	0.760	0.750	0.780	0.015	1.9	0.664	0.660	0.650	0.680	0.015	2.2
Wylecz.to	0.635	0.625	0.440	0.940	0.157	24.7	0.525	0.505	0.340	0.820	0.148	28.2	0.415	0.385	0.240	0.700	0.140	33.8

CV – coefficient of variation.

Regarding the time elapsed after consuming alcohol, almost all calculators gave more accurate results, i.e., closer to the reference value, 120 min after consumption. Only Website No. 5 obtained its best values after 60 min.

The next 3 figures compare the results from all online calculators with the reference values described by Sekula et al. [10] with the latter marked as 100%: C_max_ (Figure 3a), BAC after 60 min (Figure 3b) BAC and 120 min (Figure 3c). The data indicates that many of the results were overestimated, with M values of 139% (C_max_), 142% (60 min) and 149% (120 min). Only 1 calculator (Omnicalcalculator) showed C_max_ M <100% of the reference value; 3 websites (Omnicalculator, websites No. 8 and No. 10) showed the same trend (underestimation) after 60 min and 120 min.

The results (descriptive statistics) of the 36 tested online BAC calculators and the reference data from Sekuła et al. [10] are given in Table 4. Particularly noteworthy is the fact that the reference C_max_, 60 min and 120 min values are characterized by a large spread (0.2–0.9 g/l), and a CV ranging 25.3–33.0%. This large range may derive from the wide range of data from Sekuła et al. [10], which was obtained from 16 subjects who consumed various alcoholic beverages while fasting and after a meal. Some online calculators demonstrate very little scatter, indicated by CV <2% (7 records for C_max_, 5 for the value after 60 min and 1 for 120 min) (Table 4).

### Correlations

The correlation between the reference values and the results obtained from the online BAC calculators are presented in Table 5 (describing C_max_), Table 6 (60 min after ingestion) and Table 7 (120 min after ingestion). These tables describe websites with a CV >2% and showed only significant results based on Pearson's test (p < 0.05 or p < 0.01). Four records of significant correlations was recorded when analyzing C_max_ and 60 min results (Table 5 and 6). The highest number of significant correlations was recorded at 120 min: 11 records, of which 5 demonstrated high significance (p < 0.01) (Table 7).

**Table 5:** Correlation between the maximum concentration obtained by the online breathalyzers and the maximum reference value [10]

Website	r(X,Y)	Dependent constant Y	Slope of the dependent variable Y
Alkomaty.biz/Ilemogewypic/Website No. 7	–0.59257^**^	0.78899	–0.18147
BACscan/Aisko	0.62243^**^	0.30732	0.69272
Originalabsinthe	0.58930^**^	0.52526	0.27364
Website No. 3	0.62560^**^	0.26084	0.91618

** p < 0.01.

**Table 6:** Correlations between the online blood alcohol concentration (BAC) calculators and the reference value at 60 min after alcohol consumption [10]

Website	r(X,Y)	Dependent constant Y	Slope of the dependent variable Y
Alkomaty.biz/Ilemogewypic/Website No. 7	–0.80830^**^	0.80306	–0.24063
BACscan/Aisko	0.76930^**^	0.29446	0.83227
Originalabsinthe	0.44682^*^	0.57952	0.14218
Website No. 3	0.62916^**^	0.22033	0.73281

* p < 0.05.

** p < 0.01.

**Table 7:** Correlations between the online blood alcohol concentration (BAC) calculators and the reference value 120 min after alcohol consumption [10]

Website	r(X,Y)	Dependent constant Y	Slope of the dependent variable Y
Alkomat-online/Alkopatrol			
20 years old	0.41805^*^	0.39406	0.26721
40 years old	0.42988^*^	0.44371	0.25034
60 years old	0.42497^*^	0.51174	0.21485
Alkomaty.biz/Ilemogewypic/Website No. 7	–0.6174^**^	0.63827	–0.23367
BACscan/Aisko	0.8329^**^	0.20734	0.88874
Blokadaalkoholowa	–0.37480^*^	1.10719	–0.79626
Medme			
20 years old	0.41074^*^	0.28241	0.34879
40 years old	0.42086^*^	0.32089	0.34777
60 years old	0.42134^*^	0.36933	0.32990
Rupissed			
20 years old	0.41675^*^	0.22957	0.39979
40 years old	0.40850^*^	0.23133	0.42159
60 years old	0.39892^*^	0.23429	0.44170
Website No. 3	0.72519^**^	0.07055	0.86170
Website No. 4	–0.56960^**^	0.80868	–0.61196
Website No. 6	0.45854^**^	0.27561	0.23596
Website No. 12	–0.37490^*^	0.66038	–0.53372
Wylecz.to	0.42621^*^	0.23380	0.51818

* p < 0.05;

** p < 0.01.

For the results given in Tables 5–7, i.e., whose r-values were >0.7, graphs were additionally prepared showing the correlations in more detail. The correlations for 60 min and 120 min are given in Figure 4. No significant correlation was shown with r > 0.7 for C_max_.

A negative correlation was found between reference alcohol concentration and the alcohol concentration predicted by the website (Figure 4a); however, the difference is less significant for higher measured alcohol concentrations (>0.5 g/l) than for low concentrations: at low measured concentrations, the websites can predict more than twice the value. With an increase of 1 pt in the reference values, the mean decrease of 0.2 g/l is observed in the predicted result: Y = –0.24063 (Table 6).

In contrast, a positive correlation was found between the measured alcohol concentration and the predicted values (Figure 4b–d). Most of the predicted results are higher than the reference values. A 1-point increase in reference value is associated with the mean increase in predicted value of 0.8 g/l (Figure 4b), i.e., Y = 0.83227 (Table 6), and 0.9 g/l (Figure 4d) i.e., Y = 0.88874 (Table 7).

Figure 4c shows also a positive correlation. The Website No. 3 breathalyzer results were both overestimated and underestimated; this is most notable at a measured value of 0.4 g/l, where the greatest scatter for predicted BAC was noted (<0.3 g/l to >0.6 g/l). When the measured value increases by 1, the mean increase in the predicted result increases by 0.9 g/l.

The correlation tests served as a preliminary method for assessing the strength of the relationship between the tested breathalyzers and the measured reference values; these findings formed the basis for determining the direction of further analysis. As Pearson's correlation itself is subject to error, the method was supplemented by the Passing-Bablok test.

### Comparison and evaluation of breathalyzers with reference values

The predicted results provided by the tested breathalyzers were compared with the reference data using the Passing-Bablok test. The analysis indicated that among the breathalyzers demonstrating a significant correlation with the reference values, only 5 records were found to exhibit no significant differences with the reference method (Table 8). For these breathalyzers, the confidence interval for the slope coefficient a) has a value of 1 in its range, indicating the absence of proportional differences. Similarly, the confidence interval for the intercept, b) has a value of 0 in its range, which suggests the absence of random differences. Hence, the predicted values from the breathalyzers are comparable with those obtained using the reference method. For the online tools that were not compatible with the reference method, confidence intervals are not listed in the Table 8.

**Table 8:** The regression equations for the comparison and evaluation test of online blood alcohol concentration (BAC) prediction tools achieving significant correlations with reference data [10]

Website	Passing-Bablok regression equation	95% CI	
		a	b
Alkomat-online/Alkopatrol			
20 years old (120 min)	y = 0.291 + 0.5941 × X		
40 years old (120 min)	y = 0.3608 + 0.52 × X		
60 years old (120 min)	y = 0.4534 + 0.4062 × X		
Alkomaty.biz/Ilemogewypic/Website No. 7			
C_max_	y = 0.718 – 0.0596 × X		
60 min	y = 0.7827 – 0.1935 × X		
120 min	y = 0.56 + 0 × X		
BACscan/Aisko			
C_max_	y = 0.0149 + 1.2697 × X	0.8889–1.7407	–0.236–0.2217
60 min	y = 0.1531 + 1.125 × X		
120 min	y = 0.1406 + 1.1176 × X	0.8462–1.5556	–0.0056–0.2058
Medme (60 years old)	y = 0.2265 + 0.7817 × X	0.454–1.5	–0.02–0.3382
Odleglosci/Docelu			
60 min	y = 0.65 + 0 × X		
120 min	y = 0.5254 – 0.0438 × X		
Originalabsinthe			
C_max_	y = 0.4421 + 0.4264 × X		
60 min	y = 0.5422 + 0.2076 × X		
Website No. 3			
C_max_	y = −0.0324 + 1.5294 × X	0.84–2.3333	–0.4817–0.3152
60 min	y = 0.0516 + 1.1409 × X	0.7619–1.6957	–0.2293–0.2021
120 min	y = −0.0638 + 1.3077 × X		
Website No. 4 (120 min)	y = 0.3099 + 0.8516 × X		
Website No. 6 (C_max_)	y = 0.6117 + 0.087 × X		

C_max_ – maximum alcohol concentrations.

a – the confidence interval for the slope coefficient has a value of 1 in its range, indicating the absence of proportional differences; b – the confidence interval has a value of 0 in its range, which suggests the absence of random differences

**Figure 5.**
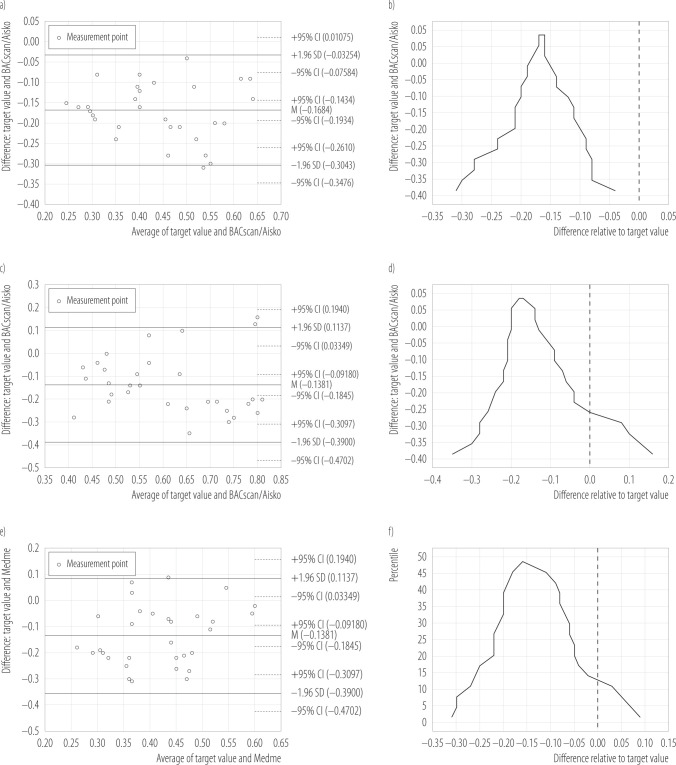
Bland-Altman and mountain plots comparing the reference method [10] with BACscan/Aisko and Medme a) Bland-Altman plot for BACscan/Aisko 120 min after alcohol consumption, b) mountain plot for BACscan/Aisko 120 min after alcohol consumption, c) Bland-Altman plot for BACscan/Aisko at C_max_, d) mountain plot for BACscan/Aisko at C_max_, e) Bland-Altman plot for Medme (60-year-old group) 120 min after alcohol consumption, and f) mountain plot for Medme (60-year-old group) 120 min after alcohol consumption

The C_max_ and 120 min results obtained by the breathalyzers BACscan and Aisko are comparable to those obtained using the reference method (Passing-Bablok test). This conclusion is confirmed by the Bland-Altman plot (Figure 5a and 5c) and the mountain plot (Figure 5b and 5d): these figures confirm that the mean differences in results provided by these breathalyzers are close to 0 for both parameters. BACscan and Aisko yield a mean overestimation of 0.168 after 120 min, and 0.138 for C_max_. However, for 60 min, both breathalyzers are subject to random errors resulting in differences the reference values.

For the 60-year-old age group, the Medme breathalyzer obtained comparable results to the reference values 120 min after alcohol consumption, as confirmed by the Bland-Altman plot and peak plot (Figure 5e and 5f). The breathalyzer overestimates the results compared to reference values by a mean score of 0.135. As no significant correlation was observed for 60 min or C_max_, the Passing-Bablok test was not performed; the same applies to the 40- and 20-year age groups.

**Figure 6.**
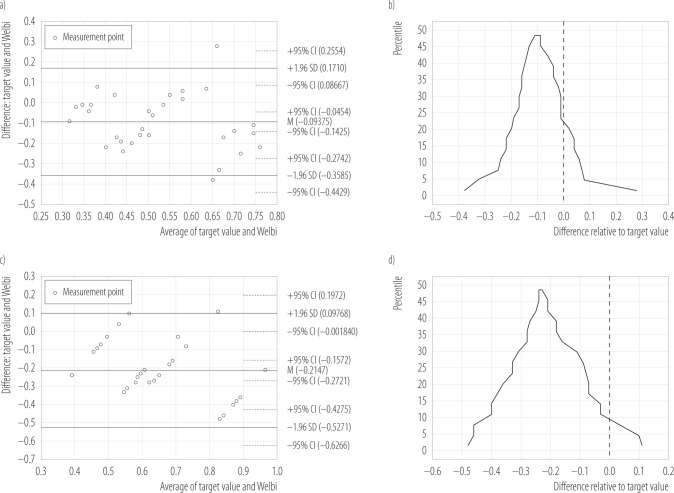
Comparison of the reference method [10] with Website No. 3: a) Bland-Altman plot 60 min after alcohol consumption, b) mountain plot 60 min after alcohol consumption, c) Bland-Altman plot for C_max_, and d) mountain plot for C_max_

The 60 min and C_max_ results obtained by the Website No. 3 tool are comparable to reference values, as confirmed by the Bland-Altman plot and mountain plot. The mean over-estimation is 0.09 for 60 min (Figure 8a and 8b) and 0.214 for C_max_ (Figure 6c and 6d). However, proportional differences can be seen at 120 min, indicating discrepancies with the reference values.

No significant differences (random or proportional) were found between the tested online breathalyzers and the measured reference values: most of the points on the Bland-Altman plots (Figure 5a, 5c, 5e, 6a, and 6c) fall within SD limits (±1.96).

The data from the remaining tested breathalyzers correlated significantly with the reference values. However, they demonstrated both proportional and random variations and cannot replace the reference method for measuring BAC.

Neither Website No. 4 nor Alko-maty (p ≥ 0.05, t-test) demonstrated significant differences with measured reference values for readings taken on an empty stomach or after a meal, based on the C_max_ values. However BACscan, Aisko and Originalabsinthe gave significantly higher values on an empty stomach compared to the values after a meal (p < 0.05), as confirmed by box-and-whisker plots. Similar results were obtained after 60 min. Additionally, BACscan, Aisko and Originalabsinthe, as well as the reference data, showed higher C_max_ values on an empty stomach than after a meal. For Website No. 4 and Alko-maty no significant differences were found between measurements taken on an empty stomach and after a meal (Table 9).

**Table 9:** A comparison of predicted blood alcohol concentration (BAC) for fasting and post-meal, depending on the time after consumption

Website	p		
	C_max_ BAC	post-meal BAC	
		after 60 min	after 120 min
Reference value	0.000026^a^	0.000000^a^	0.000000^b^
Alko-maty	1.000000^a^	0.089133^a^	0.001423^a^
Alkomaty.biz/Ilemogewypic/Website No. 7	0.000000^b^	0.000000^b^	0.000000^a^
BACscan/Aisko	0.000000^a^	0.000000^a^	0.000000^a^
Originalabsinthe	0.000000^a^	0.008050^a^	0.932194^a^
Website No. 4	1.000000^a^	0.514843^a^	0.000000^a^
Website No. 3	0.000000^b^	0.000157^b^	0.000958^a^

a T-test.

b Mann-Whitney U test.

The highest number of breathalyzers showing significant differences in predicted values was recorded after 120 min (9 records) compared to 60 min (5 records) and C_max_ (5 records). Also, at 120 min, no significant differences were demonstrated for the Originalabsinthe. Alkomaty.biz, Ilemogewypic, Website No. 7 and Website No. 4 achieved higher results after a meal compared to those obtained on an empty stomach; in contrast, Alko-maty, BACscan, Aisko and Website No. 3 produced higher results on an empty stomach than after a meal (Table 9).

The Mann-Whitney U test indicated that the Website No. 3 breathalyzer predicted higher C_max_ values on an empty stomach than after a meal, while Alkomaty.biz, Ilemogewypic, and Website No. 7 yielded higher values after a meal. For the 60 min data, the Website No. 3 breathalyzer provided higher results on an empty stomach than after a meal, while Alkomaty.biz, Ilemogewypic and Website No. 7, give higher results after a meal. After 120 min, the reference method showed higher alcohol concentrations on an empty stomach than after a meal.

### Results of the survey

A total of 103 people participated in the survey (68% women). The age of the respondents varied quite a bit: 53.4% were aged 18–25 years, 14.6% – 26–35 years, 22.3% – 36–50 years and 8.7% – 51–70 years. The survey also included 1 person >70 years. More than half (54.4%) of those surveyed were familiar with the concept of an online breathalyzer, and most (73.2%) indicated that they had used one before. The main reasons included curiosity, chosen 26 times (63.4%), followed by verification of sobriety status before driving, 15 responses (36.6%), and checking prospective calculations, 12 responses (29.3%). The least popular answer was to compare oneself with other people (9.8%, 4 responses). The majority (75.7%) of respondents declared using online breathalyzers occasionally, while 9.8% use them rarely, and 12.1% only once. One person indicated that they check their state of sobriety after every time they consume alcohol using online breathalyzers. In the next question 85.4% said they do not make decisions based on the results of online breathalyzers.

When choosing a breathalyzer, respondents paid attention primarily to the data that needs to be completed (24 votes, 58.5%), followed by popularity (15 responses, 36.6%) and the amount of data obtained (13 votes, 31.7%). The least considered factor was the layout (4 responses, 9.8%), while 2.4% do not pay attention to this aspect. The respondents most commonly used a calculator appearing highest in the list of search results (36 times, 87.8%) compared to those lower in the list (3 times, 7.3%). Five participants indicated “I have a favorite breathalyzer” (12.2%).

Respondents were then asked to mark the breathalyzers they had used. Unfortunately, the “I don't remember” option was selected 25 times (60.9%). In contrast, the most popular breathalyzer was Alkomat-online, selected 10 times (24.4%), followed by Medme (8 votes, 19.5%). Alkomaty.biz and Website No. 5 (3 times, 7.3%), and Ilemogewypic, Drinkdriving, Website No. 10, and Alkopatrol (2 times, 4.9%). Abcalkoholu, Alkotester, Kaz dypromil, BACscan, Website No. 11, Website No. 2 and Omnicalculator each received 1 mention.

The respondents also evaluated the usefulness of the breathalyzers they used: 43.9% said they were helpful, 24.4% found them not useful, 19.5% useful, 9.8% practical, and 2.4% to satisfy curiosity. Also, 75.6% of respondents said the use of online breathalyzers was uncertain, 12.2% rated them as authoritative and 9.8% were suspicious; 2.4% found the breathalyzer trustworthy.

## DISCUSSION

After consuming alcohol, the concentration in the blood is influenced by a number of factors, such as individual variation in ethanol metabolism, the fact of having eaten a meal and its composition, the type of alcoholic beverage and its percentage, the presence of chronic diseases or the consumption of medication. Generally speaking, the rate of metabolism of ethanol involves absorption, from the moment of consumption, distribution and elimination [11] and has been found to demonstrate considerable intra-personal variation [12,13]. However, online breathalyzers take limited account of this variation when determining alcohol concentration.

The BAC at a given moment is strongly influenced by the nature of the previous meal. After a meal, ethanol absorption is delayed due to slowed gastric peristalsis, slower movement of stomach contents into the duodenum, increased blood flow through the liver, and an enhanced first-pass effect [14]. Additionally, fatty foods further slow alcohol absorption from the digestive tract. Consequently, consuming alcohol on an empty stomach leads to a higher BAC in a shorter period.

The BAC is also governed to an extent by the strength of the consumed alcohol. Roberts and Robinson [15] report that alcoholic beverages with a moderate strength, i.e., ranging 10–20%, are absorbed most rapidly, while alcohol >30% demonstrates reduced absorption. Interestingly, the study suggests that carbonated beverages may accelerate alcohol absorption, although this is considered to be an individual characteristic (depends on the person).

Chronic conditions can also have an impact on blood alcohol levels, with one of the most commonly reported examples being obesity. Dunn and Tefft [16] report that a significant increase in BMI is associated with lower BAC in drivers.

In addition, different populations demonstrate variation in the activity of aldehyde dehydrogenase (ALDH), a key enzyme involved in the oxidation of aldehydes produced during alcohol metabolism. As a result, blood alcohol levels differ among ethnic groups. For example, Asian populations tend to demonstrate lower ALDH activity due to the predominance of ALDH2*2 allele encoding inactive enzyme units. In contrast, Caucasians typically exhibit full ALDH activity [17]. A similar phenomenon has been observed in individuals with alcohol addiction, where the body adapts to the constant presence of alcohol by developing metabolic tolerance and increasing the rate of elimination from the blood. This adaptation occurs through the induction of the CYP2E1 pathway in the liver, leading to increased cytochrome P450 activity, provided there is no accompanying liver damage [18].

There are widespread reports of interactions between alcohol and medication, and the concurrent use of substances such as benzodiazepines, opioids or β-blockers with alcohol has been found to have numerous side effects related to the CNS, cardiovascular system, gastrointestinal tract or hepatic function [19]. In addition, alcohol metabolism itself can also be influenced by medication. For instance, H_2_-receptor antagonists, such as cimetidine, used to treat conditions associated with excessive gastric acid secretion, increase BAC by reducing alcohol dehydrogenase (ADH) activity in the stomach [20]. Similarly, ALDH activity can also be lowered by disulfiram, which is used to treat alcohol dependence as well as various other medications such as metronidazole [19]. Ethanol metabolism can also be influenced by over-the-counter medications: acetylsalicylic acid (aspirin) has been shown to increase BAC by inhibiting ADH activity [21].

While these factors undoubtedly influence BAC, they are often not considered when assessing alcohol intoxication. The tested online breathalyzers requested easily-defined characteristics, such as sex, age, height, and body weight, but did not require information about medications being taken, ethnic background or alcohol dependence. Furthermore, most do not account for details like previously-consumed meals or body type. Another disadvantage in the online approach is also the inability to accurately determine the volume and strength of the consumed beverage: one third of the tested breathalyzers do not allow the precise amount of alcohol consumed to be entered, and >40% do not allow the percentage strength of the drink to be entered.

The authors findings indicate that the amount of information provided to breathalyzer websites does not necessarily translate into a reliable estimation of BAC. While the sites Odleglosci and Docelu took into account a range of data, *inter alia* information about sex, age, weight, height, consumed meal, and the amount and strength of alcohol, they were excluded at the beginning of the statistical analysis due to a low CV (<2%). A higher CV value is desirable in this case, as the tool should demonstrate greater variation in the results in response to individual variation and differing amounts of alcohol consumed: obtaining the same or very similar BAC may raise doubts about the reliability of the tool. On the other hand, Medme, Alkomat-online and Alkopatrol show a statistically significant correlation at a point 120 min post-consumption; they also request the most detailed data about age, meal, metabolism, body parameters, and the amount of alcohol consumed. However, the results they provide are not comparable to expected values. It is also noteworthy that high correlation coefficients (>0.7) are most commonly found in the results after 120 min of alcohol consumption, which might suggest that as time passes, the reliability of the alcohol concentrations provided by the breathalyzer increases. It was also observed that in some online breathalyzers, certain data inputs do not affect the final BAC result; for example, the same results were obtained irrespective of indicating an empty or full stomach. The mathematical model on which these breathalyzer calculations are based can therefore be considered underdeveloped, as it does not account for individual variability in BAC.

A consistent pattern observed across the majority of analyzed online BAC calculators was the systematic overestimation of alcohol concentration in comparison to reference values. This bias was evident for C_max_ as well as for measurements taken 60 min and 120 min after alcohol consumption, with mean values exceeding reference levels by approx. 39–49%. Such overestimation may result from the use of simplified pharmacokinetic models, most likely based on the Widmark formula, which assumes constant distribution and elimination parameters and does not adequately account for inter-individual variability. Additionally, the limited input data required by many calculators may lead to generalized assumptions that increase predicted BAC values. Although overestimation may appear conservative from a safety perspective, the variability and inconsistency observed between calculators indicate a lack of standardization and raise concerns about their overall reliability.

Considering the most commonly-required data requested by breathalyzer websites, it can be concluded that most use the Widmark formula [22] to calculate BAC; this approach uses a known elimination constant as well as information about body weight, sex, the amount of alcohol consumed, and the time drinking began. However, other mathematical models are also used. The Forrest formula [23] stands out by incorporating body fat content and associated BMI, while the Watson formula [24] considers total body water volume. In contrast, Posey and Mazayani [25] propose an advanced model that not only considers sex, weight, and age, but also the kinetics of alcohol absorption. An analysis of online breathalyzers by Jama, Sekuła, and Zuba [26] used the BAC Tracker International, Inc. calculator; however, the tool requires specialized knowledge needed to select an appropriate distribution coefficient.

There are efforts to create new pharmacokinetic models that better reflect real alcohol consumption patterns than the Widmark formula. Zekan et al. [27] attempted to develop a pharmacokinetic model for ethanol based on a modification of Norberg's 3-compartment model, which includes an estimation of the fraction absorbed in the portal circulation; their findings suggest that the model may be more accurate than the Widmark model, which tends to overestimate alcohol levels. In addition, an analy sis conducted by the University of Oviedo [28] Mariñas-Collado suggests that using a nonlinear model may improve the accuracy of predicting BAC.

The authors' findings suggest that the BACscan, Aisko, Website No. 3 and Medme tools produced the most reliable BAC calculations. However, these have their limitations: BACscan, Aisko and Medme demonstrate the greater convergence with the actual value than the other breath alcohol analysers, but only after a period of 120 min after alcohol consumption. In addition, Medme only gives comparable results to the measured reference values for age of 60 years old, and Website No. 3 was close to reference values at 60 min after alcohol consumption: no such correlation was observed after 120 min.

From a public health perspective, the widespread availability and use of online BAC calculators may have both beneficial and adverse consequences. On the one hand, these tools may increase awareness of alcohol consumption and its potential impact on driving ability. On the other hand, their inconsistent accuracy and lack of validation may mislead users, particularly if results are interpreted as reliable indicators of fitness to drive.

Although the present survey suggests that most users do not base their decisions solely on calculator results, a notable proportion reported using them to verify sobriety before driving. This highlights a potential risk, especially in cases where calculators underestimate BAC or where users misinterpret estimated values. Therefore, online breathalyzers should not be considered decision-support tools in safety-critical situations, and their limitations should be clearly communicated to users.

## CONCLUSIONS

Online breathalyzers offer easy accessibility, and can serve as a quick tool for estimating BAC; however, users should be aware of their limitations. Most of the tested breathalyzers report inflated alcohol concentration levels compared to the reference method, which suggests they are not entirely reliable. One particular risk is that they may falsely indicate sobriety.

According to questionnaire results, online breathalyzers are mainly used by young adults, with curiosity being the primary motivation. They are not suitable for legal or safety-critical decision-making. Only breathalyzers used for law enforcement purposes (i.e., certified breath analyzers) provide reliable BAC assessments: any final verification of sobriety should be performed using a certified breathalyzer.

### Limitations of the study

This study has some limitations. The relatively small reference sample (N = 16) limits the statistical power of the analysis and does not fully reflect inter-individual variability. In addition, only 36 online BAC calculators were assessed, so the findings should be interpreted only for the tools included in this study.
